# Child-Robot Interactions for Second Language Tutoring to Preschool Children

**DOI:** 10.3389/fnhum.2017.00073

**Published:** 2017-03-02

**Authors:** Paul Vogt, Mirjam de Haas, Chiara de Jong, Peta Baxter, Emiel Krahmer

**Affiliations:** Tilburg Center for Cognition and Communication, Tilburg UniversityTilburg, Netherlands

**Keywords:** social robots, second language tutoring, education, child-robot interaction, robot assisted language learning

## Abstract

In this digital age social robots will increasingly be used for educational purposes, such as second language tutoring. In this perspective article, we propose a number of design features to develop a child-friendly social robot that can effectively support children in second language learning, and we discuss some technical challenges for developing these. The features we propose include choices to develop the robot such that it can act as a peer to motivate the child during second language learning and build trust at the same time, while still being more knowledgeable than the child and scaffolding that knowledge in adult-like manner. We also believe that the first impressions children have about robots are crucial for them to build trust and common ground, which would support child-robot interactions in the long term. We therefore propose a strategy to introduce the robot in a safe way to toddlers. Other features relate to the ability to adapt to individual children’s language proficiency, respond contingently, both temporally and semantically, establish joint attention, use meaningful gestures, provide effective feedback and monitor children’s learning progress. Technical challenges we observe include automatic speech recognition (ASR) for children, reliable object recognition to facilitate semantic contingency and establishing joint attention, and developing human-like gestures with a robot that does not have the same morphology humans have. We briefly discuss an experiment in which we investigate how children respond to different forms of feedback the robot can give.

## Social Robots for Second Language Tutoring

Given the globalization of our society, it is becoming increasingly important for people to speak multiple languages. For instance, the ability to speak foreign languages fosters people’s mobility and increases their chances for employment. Moreover, immigrants to a country need to learn the official host language. Since young children are most flexible at learning languages, starting second language (L2) learning in preschool would provide them a good opportunity to acquire the second language more fluently at a later age (Hoff, 2013).

One trend in the digital age of the 21st century is that technologies are being developed for educational purposes, including technologies to support L2 tutoring. There exist many forms of digital technologies for PCs, laptops or tablet computers that support second language learning, although there is little evidence about their efficacy (Golonka et al., 2014; Hsin et al., 2014). While children can benefit from playing with such technologies, these systems lack the situated and embodied interactions that young children naturally engage in and learn from (Glenberg, 2010; Leyzberg et al., 2012). Social robots represent an emerging technology that provides situatedness and embodiment, and thus have potential benefits for educational purposes. In essence, social robots are autonomous physical agents, often with human-like feature, that can interact socially with humans in a semi-natural way for prolonged periods of time (Dautenhahn, 2007). The use of social robots, in comparison to more traditional digital technologies, allows for the development of tutoring systems more akin to human tutors, especially with respect to the situated and embodied social interactions between child and robot. Thus, this offers the opportunity to design robots such that they interact in a way that optimizes the child’s language learning.

Recently, an increasing interest has emerged to develop social robots to support children with learning a second language (Kanda et al., 2004; Belpaeme et al., 2015; Kennedy et al., 2016). While a social robot cannot provide tutoring to the level humans can, recent studies suggest that using social robots can result in an increased learning gain compared to digital learning environments for tablets or computers (Han et al., 2008; Leyzberg et al., 2012). It is, however, unclear why this is the case. Perhaps the physical presence of the robot draws the attention of children for longer periods of time, but the embodiment and situatedness of the learning environment perhaps also helps the children to ground the language more strongly than interactions with virtual objects do.

While there is a fair body of research on robot tutors, a comprehensive description of the design features for a second language robot tutor based on what is known about children’s language acquisition is lacking. What are the design features of child-robot interactions that would support second language learning? And, to what extent can these interactions be implemented in today’s social robot technologies? In this perspective article, we try to answer these questions based on theoretical accounts from the literature on children’s language acquisition in combination with our own experiences in designing a tutor robot.

## Designing Child-Robot Interactions

In our project, we aim to design a digital learning environment in which preschool children interact one-on-one with a social robot that supports either their learning of English as a foreign language, or the school language for those children who have a different native language (Belpaeme et al., 2015). In particular, the project aims to develop a series of tutoring sessions revolving around three increasingly complex domains (numbers, spatial relations and mental vocabulary). In each session, the child will engage with the robot (a Softbank Robotics NAO robot) in a game-like scenario focusing on learning a small number of target words. The contextual setting is generally displayed on a tablet computer that occasionally also provides some verbal support, however, the robot acts as the interactive tutor. Below we discuss the design features and considerations that we believe are crucial to design a successful tutoring system.

### Peer-Like Tutoring

One of the first questions that comes up when designing a robot tutor is whether the robot should take the role of a teacher or a peer. Research on children’s language acquisition has demonstrated that children learn more effectively from an adult who can use well-defined pedagogical methods for teaching children using clear directions, explanations and positive feedback methods (Matthews et al., 2007). However, designing and framing the robot as an adult tutor has the disadvantage that children will form expectations about the robot’s behavior and proficiency that cannot be met with current technology (Kennedy et al., 2015). Due to technological limitations of the robot and underlying software, communication breakdowns are more likely to occur than with a human. For a peer robot introduced as a fellow language learner, breakdowns in communication are more acceptable. Moreover, interacting with robots acting as peers is conceived as more fun (Kanda et al., 2004), allows for learning-by-teaching (Tanaka and Matsuzoe, 2012) and has a proven to be efficient in teaching children how to write (Hood et al., 2015). Furthermore, there is some evidence that children’s learning can benefit from interacting with peers (Mashburn et al., 2009). Given these considerations, we believe it is desirable to frame or introduce the robot as a peer and friend, yet design its interactions insofar possible based on pedagogically well-established strategies to scaffold language learning.

### First Impressions

To implement effective tutoring, the robot needs to interact with children in multiple sessions, so they have to be motivated to engage in long-term interactions with the robot. Establishing common ground between child and robot can contribute to this (Kanda et al., 2004), but first impressions to establish trust and rapport are also crucial (Hancock et al., 2011).

Despite the wealth of studies regarding the introduction of entertainment robots as toys to children (e.g., Lund, 2003), surprisingly little research has been conducted on designing protocols on how to introduce a robot tutor to a group of preschool children. Fridin (2014) presents one exception, and found that introducing a robot tutor to children in group sessions improved subsequent interactions compared to introducing the robot to children in individual sessions. Another study by Westlund et al. (2016) found that the way a robot is framed, either as a machine or a social entity, affected the way children later engaged with the robot. They concluded that introducing the robot as a machine could create a more distant relation between child and robot, thus reducing acceptance. We therefore decided to frame the robot in our project as a social playmate for the children and introduced the robot in a group session. However, the NAO robot is slightly taller and more rigid than the fluffy huggable Tega robot, which Westlund et al. (2016) used, and we observed that some 3-year-old children were somewhat intimidated by the NAO robot on their first encounter. Such a first impression of the robot could reduce the trust that the child had for the robot, which could negatively affect their willingness to interact with the robot in the short-term, but also in the long-term. To develop a successful first encounter and to build trust between the child and robot, we designed the following strategy for introducing the robot to 3-year-old children at their preschool.

Pilot studies revealed that some children got anxious when the robot was introduced and then suddenly started to move. To familiarize children prior to their first encounter with the robot, it is therefore advisable to prepare them well. For our study, we sent coloring pages of the robot to the preschools during recruitment and asked the pedagogical assistants to talk a little bit about the robots to the children. About 1 week before the experimental trials, the experimenters introduced the robot in class during their daily “circle time”, as this provided a safe and familiar environment with the whole group in which the pedagogical assistants usually introduce new topics or new activities. One experimenter first introduced the robot by telling a story about Robin, the name of our robot, using a makeshift picture book. In this story we explained the similarities and dissimilarities between the robot and children to construct the type of common ground considered to have a positive effect on the learning outcome (Kanda et al., 2004). For example, we told that Robin enjoys dancing and wants to meet new friends, and even though he does not have a mouth and because of that cannot smile, he can smile using his eye LEDs.

After this story, another experimenter entered the room with the robot while it was actively looking at faces to provide an animate feeling. The robot introduced itself with a small story about itself and by performing a dance in which the children were encouraged to participate. The end of the circle time consisted of getting a blanket for the robot so it could “sleep”. This introduction was repeated later on the days we conducted the experiment in one-on-one sessions. While by then most children were comfortable interacting with the robot, some were still timid and anxious. To encourage these children to feel comfortable, one of the experiment leaders would sit next to the child during the warm-up phase of the experiment and motivate the child to respond to the robot when necessary until the child was sufficiently comfortable to interact with the robot by herself/himself. We found that the younger 3-year olds required more support from the experimenters than the older 3-year olds (Baxter et al., 2017). Although we are still analyzing the experiments, preliminary findings suggest that our introduction helped children to build trust and common ground with the robot effectively.

### Temporal Contingency

Research has shown that it is crucial for children’s language development that their communication bids are responded to in a temporally contingent manner (Bornstein et al., 2008; McGillion et al., 2013). This, however, faces a technological challenge. While adults tend to take over turns very rapidly, robots require relatively long processing time to produce a response. Nevertheless, in our first experiment (de Haas et al., 2016), we observed that children were at first surprised by the delayed responses, but quickly adapted to the robot and waited patiently for a response. Perhaps this is because children also require longer than adults to take turns (Garvey and Berninger, 1981) and having framed the robot as a peer children made the delays more plausible or expected. Nevertheless, while a lag in temporal contingency may not harm the interaction with children, it may harm learning. One way to remedy this may be to have the robot start responding by providing a backchannel signal, such as “uhm” to indicate the robot is (still) taking his turn, but requires more time to process (Clark, 1996).

### Semantic Contingency

Robots should not only respond to children in a timely fashion, but also in a semantically contingent fashion (i.e., consistent with the child’s focus of attention), as this too has a positive effect on children’s language acquisition (Bornstein et al., 2008; McGillion et al., 2013). For instance, research has shown that by responding in a semantically contingent manner, either verbally or by following children’s gaze, (joint) attention is sustained for a longer duration (Yu and Smith, 2016), allowing children to learn more about a situation. To achieve semantically contingent responses, the robot should be able to understand the child’s communication bids, construct joint attention with the child, or at least identify what the child is attending to. Monitoring children’s behavior and establishing joint attention are therefore considered crucial for designing a successful robot tutor.

### Monitoring Children’s Behavior

To understand children’s communication bids, as well as to test their pronunciation of the L2, it is important that the robot be equipped with well-functioning automatic speech recognition (ASR). However, the performance of state-of-the-art ASR for children is still suboptimal, especially for preschool-aged children (Fringi et al., 2015; Kennedy et al., 2017). Reasons for this include that children’s pronunciation is often flawed and that their speech has a different pitch than adults. Moreover, relatively little research has been carried out in this domain and not much data exist to train ASR on. While it can be expected that the performance of ASR for children will improve in the not too distant future (Liao et al., 2015), until then alternative strategies need to be developed that do not (exclusively) rely on ASR.

In our project, we explore various strategies to achieve this, both based on monitoring non-verbal behaviors of the children and focusing on comprehending rather than producing L2. The first strategy relies on providing children tasks they have to perform in the learning environment, such as placing “a toy cow behind a tree” when teaching spatial language. This, however, requires the visual object recognition on the robot to work well, which is only the case when the scene contains a limited set of distinctively recognizable objects, such as distinctly colored objects (Nguyen et al., 2015). A potential solution explored in our project is to use objects with build-in RFID sensors that can be tracked automatically. The second solution we explore is to use a touch screen tablet that displays scenes the child can manipulate, which not only has the advantage of avoiding the problem of object recognition, but also allows us to control the robot’s responses and vary the scenes in real time. A downside, however, is that it takes away the 3-dimensional physical aspect of embodied cognition that would help the children to better entrench what they learn (Glenberg, 2010). Currently, experiments are underway to investigate the effect of using real vs. virtual objects. These solutions not only aid in understanding the child’s communication bids, it also helps in identifying their attention and can thus contribute to establishing joint attention.

### Joint Attention and Gestures

**Figure 1:**
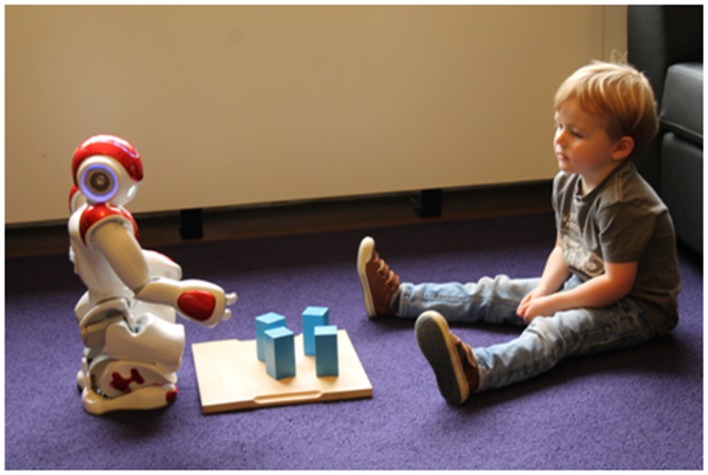
**NAO pointing to a block with three fingers**. (Note that written, informed consent was obtained from the parents of the child for the publication of this image).

Joint attention, where interlocutors attend on the same referent, is a form of social interaction that has been shown to support children’s language learning (Tomasello and Farrar, 1986). One way to establish joint attention with a child is to guide their attention to a referent using gestures, such as pointing or iconic gestures. The ability to produce gestures in the real world is potentially one of the main advantages of using physical robots as opposed to virtual agents, who may have a harder time to establish joint attention. However, many robots’ physical morphologies do not correspond one-to-one to the human body. Hence, many human gestures cannot be translated directly to robot gestures. For instance, the NAO robot that we use in our research has a hand with three fingers that cannot be controlled independently, so index finger pointing cannot be achieved (see Figure 1). Will children still recognize NAO’s arm extension as a pointing gesture? And if so, will they be able to identify the object the robot refers to? We are currently running an experiment to investigate how NAO’s pointing gestures are perceived, and preliminary findings show that participants have difficulty identifying the referred object on a small tablet screen. Similar issues arise when developing other gestures. One of the other non-verbal behaviors we are using is the coloring of NAO’s eye LEDSs to indicate the robot’s happiness as a form of positive feedback, since the robot cannot smile with its mouth.

### Feedback

Feedback, too, is an interactional feature known to help language learning (Matthews et al., 2007; Ateş-Şen and Küntay, 2015). The question is how should the robot provide feedback, such that it is both pleasant and effective for learning? While adults provide positive feedback explicitly, they usually provide negative feedback implicitly by reformulating children’s errors in the correct form. In child-child interactions, however, Long (2006) found that there was a clear advantage in learning from explicit negative feedback (e.g., by saying “no, that’s wrong, you need to say ‘he ran”’) when compared to reformulating feedback (the learner says “he runned” and the teacher reacts with “he ran”).

To investigate how children experience feedback from a peer robot, we carried out an experiment among 85 3-year-old Dutch-speaking children at preschools in Netherlands (de Haas et al., 2016, 2017). In this experiment, the children interacted with a NAO robot during which they received a short lesson on how to count from 1 to 4 in English. After a short training phase, in which the children were presented with the four counting words twice in relation to body parts and wooden blocks, they were given instructions by the robot to pick up a given number of blocks. While the instructions were given in their native language, the numbers were uttered in English. In response to the child’s ability to achieve the task, the robot provided feedback. The experiment followed a between-subjects design with three conditions: adult-like feedback (explicit positive and implicit negative), peer-like feedback (no positive and explicit negative) and no feedback. We did not find significant differences in learning gain between the conditions, probably because the target words were insufficiently often repeated. However, we explored the way in which the children engaged with the robot after they received feedback and we found that children looked less often at the experimenter in the feedback conditions than in the no feedback condition. Further analyses are carried out to evaluate how the children responded to the various forms of feedback to find out what type of feedback would be most effective for achieving both acceptable and effective tutoring interactions.

### Zone of Proximity and Adaptivity

Finally, from a pedagogical point of view it is desirable that the interactions between child and robot be sufficiently challenging and varied so that the child has a target to learn from, but at the same time interactions should not be too difficult, because that may frustrate the child causing it to lose interest in the robot (Charisi et al., 2016). In other words, the robot should remain in Vygotsky’s Zone of Proximity that supports an effective learning environment (Vygotsky, 1978). In order to achieve this, the robot should be able to keep track of the children’s advancements in language learning and perhaps their emotional states during the tutoring sessions, and adapt to these. While the former can be monitored as discussed previously, it may be possible to detect emotional states known to influence learning (e.g., concentration, confusion, frustration and boredom) using methods from affective computing (D’Mello and Graesser, 2012). Using this type of information, it is possible to adapt the tutoring sessions by either reducing or increasing the number of repetitions, and/or change the subject (Schodde et al., 2017).

## Conclusion

This perspective article presented some design features that we consider crucial for developing a social robot as an effective second language tutor. We believe the robot is most effective when it is framed as a peer, i.e., as a fellow language learner and playmate, but that is designed to use adult-like interaction strategies to optimize learning efficacy. In order to establish common ground and trust to facilitate long-term interactions, we consider it essential that the robot be introduced with appropriate care on the first encounter. As an example, we outlined our strategy for introducing a robot to preschool children. Interactions between child and robot should be contingent and multimodal, and provide appropriate forms of feedback. We argued that the robot should remain within Vygotsky (1978) Zone of Proximal Development and thus should adapt to the individual level of the child.

We also discussed some technical challenges that need to be solved in order to implement contingent interactions; the most important of which we believe is ASR, which presently does not work well for children’s speech. While various technical challenges still remain, we expect that social robots will provide effective digital technologies to support second language development in the years to come.

The present list of design features covers many aspects that need to be considered when developing a tutor robot, but it is not yet comprehensive. One aspect that has not been covered, for instance, concerns the design of robots for children from different cultures, which could require different design choices (Shahid et al., 2014). For example, in some cultures education is more teaching-centered (Hofstede, 1986) and thus designing the tutor as a peer robot may be less effective or acceptable (Tazhigaliyeva et al., 2016). Concluding, this perspective article offers only a first step towards a comprehensive list of design features for tutor robots and additional research is needed to complete and optimize the list.

## Ethics Statement

The Research Ethics Committee of Tilburg School of Humanities approved this study, and the parents of all participating children gave written informed consent in accordance with the Declaration of Helsinki.

## Author Contributions

PV, MH and EK designed the conceptual aspects of the article; PV, MH, CJ and PB carried out the literature review; PV, EK and MH designed the feedback study; MH, CJ and PB designed the introduction study; MH, CJ and PB carried out the studies; PV and MH wrote the article; CJ, PB and EK revised the article critically.

## Funding

This work has been supported by the EU H2020 L2TOR project (grant 688014). CJ and PB thank the research trainee program of the Tilburg School of Humanities for their support.

## Conflict of Interest Statement

The authors declare that the research was conducted in the absence of any commercial or financial relationships that could be construed as a potential conflict of interest.
